# Applying a Deep Learning Model for Total Kidney Volume Measurement in Autosomal Dominant Polycystic Kidney Disease

**DOI:** 10.3390/bioengineering11100963

**Published:** 2024-09-26

**Authors:** Jia-Lien Hsu, Anandakumar Singaravelan, Chih-Yun Lai, Zhi-Lin Li, Chia-Nan Lin, Wen-Shuo Wu, Tze-Wah Kao, Pei-Lun Chu

**Affiliations:** 1 Department of Computer Science and Information Engineering, Fu Jen Catholic University, New Taipei City 24205, Taiwan; alien@csie.fju.edu.tw (J.-L.H.);; 2 Graduate Institute of Applied Science and Engineering, Fu Jen Catholic University, New Taipei City 24205, Taiwan; ananthagopi003@gmail.com; 3 Department of Internal Medicine, Fu Jen Catholic University Hospital, Fu Jen Catholic University, New Taipei City 24205, Taiwan; 4 Department of Medical Imaging, Fu Jen Catholic University Hospital, New Taipei City 24352, Taiwan; 5 School of Medicine, College of Medicine, Fu Jen Catholic University, New Taipei City 24205, Taiwan; 6 PhD Program in Pharmaceutical Biotechnology, Fu Jen Catholic University, New Taipei City 24205, Taiwan

**Keywords:** autosomal dominant polycystic kidney disease (ADPKD), total kidney volume (TKV), deep learning, artificial intelligence (AI)

## Abstract

Background: Autosomal dominant polycystic kidney disease (ADPKD) is the most common hereditary renal disease leading to end-stage renal disease. Total kidney volume (TKV) measurement has been considered as a surrogate in the evaluation of disease severity and prognostic predictor of ADPKD. However, the traditional manual measurement of TKV by medical professionals is labor-intensive, time-consuming, and human error prone. Materials and methods: In this investigation, we conducted TKV measurements utilizing magnetic resonance imaging (MRI) data. The dataset consisted of 30 patients with ADPKD and 10 healthy individuals. To calculate TKV, we trained models using both coronal- and axial-section MRI images. The process involved extracting images in Digital Imaging and Communications in Medicine (DICOM) format, followed by augmentation and labeling. We employed a U-net model for image segmentation, generating mask images of the target areas. Subsequent post-processing steps and TKV estimation were performed based on the outputs obtained from these mask images. Results: The average TKV, as assessed by medical professionals from the testing dataset, was 1501.84 ± 965.85 mL with axial-section images and 1740.31 ± 1172.21 mL with coronal-section images, respectively (*p* = 0.73). Utilizing the deep learning model, the mean TKV derived from axial- and coronal-section images was 1536.33 ± 958.68 mL and 1636.25 ± 964.67 mL, respectively (*p* = 0.85). The discrepancy in mean TKV between medical professionals and the deep learning model was 44.23 ± 58.69 mL with axial-section images (*p* = 0.8) and 329.12 ± 352.56 mL with coronal-section images (*p* = 0.9), respectively. The average variability in TKV measurement was 21.6% with the coronal-section model and 3.95% with the axial-section model. The axial-section model demonstrated a mean Dice Similarity Coefficient (DSC) of 0.89 ± 0.27 and an average patient-wise Jaccard coefficient of 0.86 ± 0.27, while the mean DSC and Jaccard coefficient of the coronal-section model were 0.82 ± 0.29 and 0.77 ± 0.31, respectively. Conclusion: The integration of deep learning into image processing and interpretation is becoming increasingly prevalent in clinical practice. In our pilot study, we conducted a comparative analysis of the performance of a deep learning model alongside corresponding axial- and coronal-section models, a comparison that has been less explored in prior research. Our findings suggest that our deep learning model for TKV measurement performs comparably to medical professionals. However, we observed that varying image orientations could introduce measurement bias. Specifically, our AI model exhibited superior performance with axial-section images compared to coronal-section images.

## 1. Introduction

Autosomal dominant polycystic kidney disease (ADPKD) is one of the most common inherited kidney diseases, with a prevalence rate ranging from 1/400 to 5/10,000 [1,2,3]. ADPKD is primarily caused by mutations in Polycystin 1 (*PKD1*) or Polycystin 2 (*PKD2*). ADPKD is characterized by the gradual development of renal cysts and the enlargement of these cysts over time. As the number and size of renal cysts increase, there is a progressive enlargement of both kidneys, typically with an average annual growth rate ranging from 2% to 5% [4]. This continuous growth of renal cysts contributes to a gradual decline in renal function, ultimately leading to end-stage renal disease (ESRD). It is estimated that nearly half of ADPKD patients progress to ESRD by the age of 60 years and need lifelong dialysis or kidney transplantation [5]. Tolvaptan has been shown to slow down the progression of renal cysts in both the early and late stages of ADPKD, but it is not a cure [6,7]. Clinically, total kidney volume (TKV) inversely correlates with ADPKD patients’ renal function, and it has been utilized as an indicator and predictor of renal outcome in ADPKD [8]. Thus, TKV can also be used to evaluate the effectiveness of medical treatment. Magnetic resonance imaging (MRI), computed tomography (CT), and ultrasound are all viable options for estimating total kidney volume (TKV), with each imaging modality offering its own set of advantages and drawbacks [9]. TKV measurement using MRI provides exceptionally high reliability with minimal variation [10]. The Consortium for Radiologic Imaging Studies of Polycystic Kidney Disease (CRISP) also suggests that MRI is the most effective method for detecting variations in kidney size over short time intervals, although it requires manual renal tracing [11]. Consequently, considering factors such as accuracy, radiation exposure, and the risk of contrast-associated nephropathy, T2-weighted MRI may be the preferred choice despite its relatively higher cost.

There are several methods being utilized to measure TKV, including manual planimetry tracing [12], the stereological method [13], the mid-slice method [14], and the ellipsoid equation [15]. Among them, manual planimetry tracing is the easiest and the most straightforward method, but it relies on the expertise and experiences of medical professionals. Additionally, it is a tedious, time-consuming process that very likely leads to measurement bias caused by human error. The stereological method has an accuracy comparable to that of planimetry tracing, but the analytic time is the drawback as well [9]. The mid-slice method is based on the assumption that the kidney shape is ellipsoidal, and stereological determination is its first step. Because of its assumption, the mid-slice method also leads to measurement bias [9,14]. The ellipsoid equation has been demonstrated to have poor repeatability and reproducibility [16]. Collectively, an efficient, accurate, and minimal human error-prone method is eagerly needed, such as semi-automated or fully automated methods [17,18,19]. Currently, the semi-automated method is more applicable in clinical practice [20,21].

Artificial intelligence (AI) serves as a supportive tool in healthcare, particularly through the application of deep learning methodologies. These techniques have been utilized across a spectrum of image-based applications to assess the presence and severity of diseases [22,23]. The implementation of a deep learning-based approach has greatly improved the performance and efficiency of organ segmentation strategies, particularly in kidney segmentation and subsequent TKV measurement [24]. In order to acquire a reference or training dataset for TKV measurement, it was often necessary to establish manual segmentation [25]. Convolutional neural networks (CNNs) represent a network architecture within the realm of deep learning, specifically tailored to tackle challenges in medical image analysis. CNNs have been successfully employed in medical image segmentation, and leveraging this approach, kidney segmentation based on MRI images has been achieved through the utilization of a reconstructed three-dimensional (3D) U-Net model [26]. The U-Net-based method has been previously implemented into an automated system to segment kidneys of chronic kidney disease [27]. An alternative approach of automated segmentation of kidneys and TKV measurement adapted a combination of VGG16 and a CNN to analyze CT images and compute TKV [28]. However, the training period for the 3D-based segmentation model required a considerable computing resource and was time-consuming. Bevilacqua et al. utilized the R-CNN to identify the region of interest containing the kidney, followed by applying a CNN for semantic segmentation to precisely define the kidney boundary [29]. In this pilot study, we developed a novel semi-automated deep learning model for measuring TKV in patients with ADPKD using MRI images. We analyzed both axial- and coronal-section MRI images and found that axial images provided more accurate and reliable results for TKV calculation compared to coronal images.

## 2. Methods and Materials

**Figure 1:**
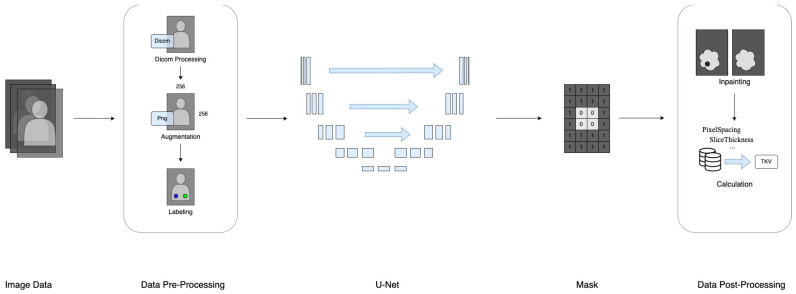
The research framework of data processing.

This section provides a comprehensive description of the datasets utilized and the deep learning methodology employed, covering specifications, format, data collection procedures, pre-processing techniques, and data labeling. Additionally, a flowchart illustrating the study’s progression from data preparation to TKV estimation is included in Figure 1.

### 2.1. Data Collection and Specifications

The MRI images were collected from the Fu-Jen Catholic University Hospital, with Institutional Review Board approval obtained for the research study (IRB No. FJUH109022). The data were in DICOM format (.dcm), and kidney MRI scans were conducted using the T2-weighted mode, encompassing both axial and coronal sections. MRI image sets from a total of 40 participants, 30 randomly selected ADPKD patients, and 10 randomly selected healthy participants were obtained for analysis. The inclusion criteria were as follows:

1. Patients with polycystic kidney disease who are at least 20 years old;
2. High-quality MRI images as determined by a radiologist.

Any patients who did not meet these criteria were excluded from the MRI image export.

All potentially identifying information has been removed to ensure participant anonymity. Therefore, no informed consent was required, as approved by the IRB committee.

### 2.2. Data Pre-Processing

After obtaining the raw data, further pre-processing was conducted to prepare the data for subsequent training and testing. Initially, images were extracted from the source DICOM files. DICOM tags containing relevant data were then extracted from these DICOM files. Subsequently, essential information such as pixel spacing (the area of each pixel), slice thickness (the thickness of each image slice), spacing between slices (the distance between each image slice), as well as the number of rows and columns of the image were retrieved from the DICOM tags.

### 2.3. Data Augmentation

Given the limited availability of medical data, particularly for rare diseases, and the challenge of accessing medical images, training a deep learning model with a small dataset can lead to overfitting. To mitigate this bias, we implemented data augmentation techniques to increase the dataset volume. This involved applying operations such as rotation, zoom, shift, and flip to the image data based on specified parameters. These operations generated additional images with variations from the original dataset. To maintain similarity between the generated and source images, we carefully controlled the parameter settings. Specifically, we set the rotation parameter to 5 degrees while both the shift and zoom parameters were set to 10%. We did not utilize horizontal or vertical flip settings to minimize discrepancies during training. We split the dataset of 1483 axial images into training and validation sets, with 1186 images for training and 297 for validation, following an 80:20 ratio. To improve model generalization, we applied data augmentation using Keras’s ImageDataGenerator, which randomly transforms the training images based on specified parameters. The model was trained for 500 epochs, with a new set of augmented images generated during each epoch. This random augmentation process exposed the model to different variations of the training images, allowing it to learn from a broader range of transformations. The same approach was applied to the coronal training set.

### 2.4. Data Labeling and Management

**Figure 2:**
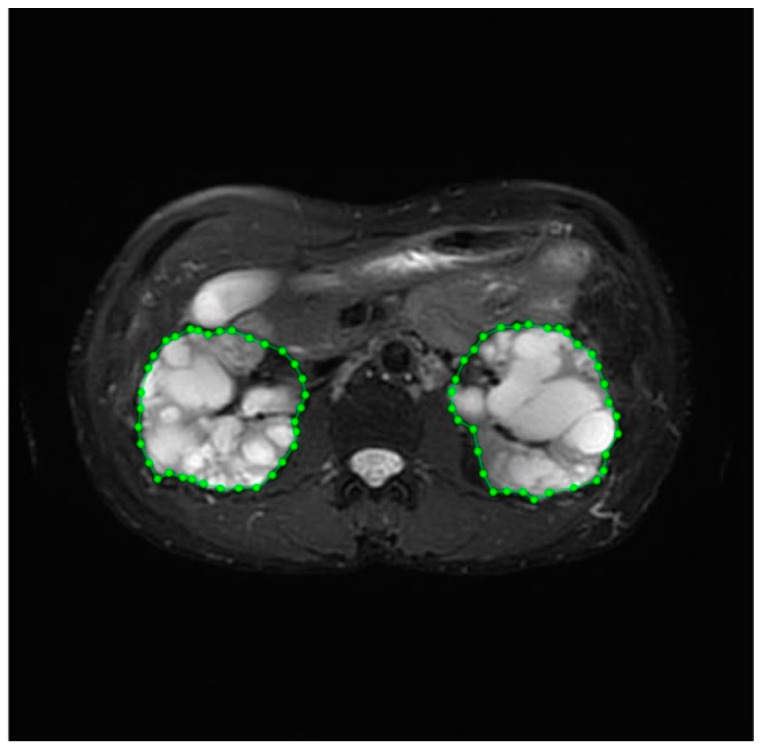
The labeling process of kidney volume.

Image annotation, a complex and time-consuming process, was crucial for improving the reliability of the data. This task was performed by experienced medical professionals at the Department of Medical Imaging, Fu Jen Catholic University Hospital. Therefore, our dataset underwent meticulous pre-processing techniques before use. The annotation process included identifying the position and boundaries of the kidneys to generate mask images for image segmentation. Labelme (http://labelme.csail.mit.edu/Release3.0/, accessed on 17 July 2020), an image annotation tool, was utilized for this purpose. When using Labelme, there are five options for creating annotations: “Create Polygons”, “Create Rectangle”, “Create Circle”, “Create Line”, and “Create Point.” Of these, the “Create Polygons” option is the most suitable for describing objects with arbitrary shapes. We utilized the “Create Polygon” option with clicks to delineate paths on the required portions of the images. The left and right kidneys were accurately circled and saved as labeled image files. Consequently, a comprehensive dataset was successfully curated for the training procedure. The labeling process is illustrated in Figure 2. Prior to training, the input image and mask are normalized to a range of [0, 1] by scaling their pixel values down through division by 255. The model was trained using a paired dataset comprising original images and corresponding mask images. The model’s input consisted of the original medical images, while the output comprised mask images with the kidney region delineated.

### 2.5. Deep Learning Model

#### U-Net

**Figure 3:**
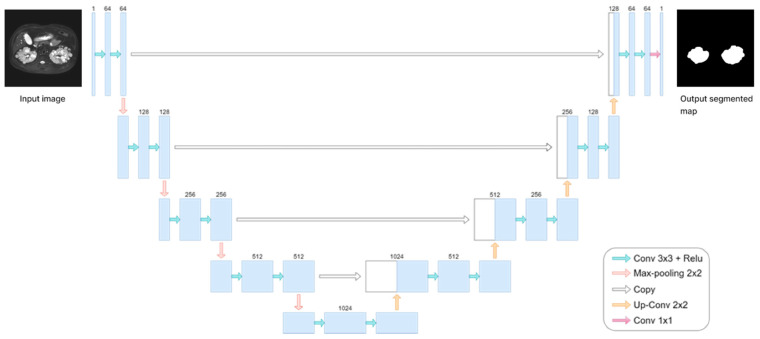
The framework of the U-net model.

In our pilot study, we encountered challenges associated with obtaining medical image data from patients with a relatively rare disease, similar to the situation reported by Ronneberer et al. [30]. Consequently, we adopted the U-net model for kidney segmentation based on the methodology outlined in Ronneberer et al.’s report, with minor modifications, and computed TKV based on the segmentation results. As shown in Figure 3, the U-net model, a fully convolutional network, consists of both down-sampling and up-sampling blocks, featuring a contracting path on the left and an expansive path on the right. Briefly, our U-net model employed binary image segmentation with “padding = same” to prevent slight degradation during each convolution, as observed in Ronneberer et al.’s report. Additionally, images sized 256 × 256 were retrieved and processed to ensure uniformity in size. The model processes 2D images as input and generates 2D images (masks) as output. We utilized the He-initializer for weight initialization instead of random weight initialization, which contributed to improved training efficiency and performance [31]. We set the Batch Size to 10 and incorporated dropout with a rate of 0.2 at the end of the contracting path to mitigate overfitting. ReLU activation was used in every convolutional layer except the last one, where sigmoid activation was employed. Additionally, we applied the warm-up exponential decay technique to gradually increase the learning rate before decaying it, ensuring smooth training. A Dice coefficient-based loss function was utilized for performance evaluation. The output image of this model ranged between values of 0s and 1s.

### 2.6. Loss Function

During the training procedure, we evaluated our results using the Dice Similarity Coefficient (DSC) loss function. The DSC, also known as the Sørensen-Dice coefficient, is a statistic used to quantify the similarity between two sets. In the context of image segmentation or medical image analysis, the DSC is often employed to assess the agreement between the predicted and ground truth segmentations. The DSC is a value between 0 and 1, where 0 indicates no overlap between the sets (complete dissimilarity), and 1 indicates perfect overlap (complete similarity). The calculation of the DSC is shown as follows: DSC = 2TP/(2TP + FP + FN), where TP represents true positive, FP represents false positive, and FN represents false negative.

### 2.7. Optimizer

During the training process, the choice of optimizer and loss function played pivotal roles. Initially, the original U-net paper employed the Stochastic Gradient Descent (SGD) with Momentum [30]. The SGD updated weights and biases based on the gradients. However, using the SGD often led to slow convergence and oscillation, complicating the model’s ability to reach the global minima. Addressing these issues, the inclusion of momentum control helped mitigate oscillation and expedite convergence. In our study, we opted for the Adaptive Moment Estimation (Adam) optimizer instead of the SGD. Adam is a widely used optimizer in deep learning, incorporating elements of Root Mean Squared Propagation (RMSprop) and Momentum. The RMSprop automatically adjusted the learning rate according to the inverse-square value of the past gradient and rectified the problem of the excessive drop of learning rate in the Adaptive Gradient Algorithm (Adagrad). Based on the gradient, Momentum accumulates the proportion of the previous parameter value to the current parameter value, which enhanced stability during early training by implementing a bias-correction strategy to reduce the impact of initialization parameters. Overall, Adam demonstrated superior convergence speed and results compared to methods such as SGD, AdaGrad, and RMSprop.

### 2.8. Data Post-Processing: Inpainting and Volume Calculation

**Figure 4:**
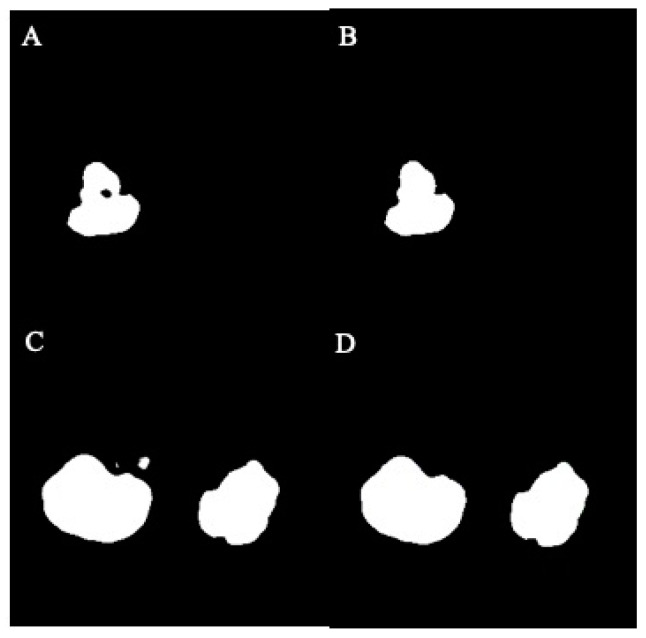
The effect of hole-filling and despeckle processes in example images: (**A**) before filling, (**B**) after filling, (**C**) before despeckle, (**D**) after despeckle.

In this study, the U-Net model processed original MRI images and generated mask images representing the target regions. These mask images were categorized into two groups: normal masks and damaged masks. The damaged masks encompassed masks with defects, holes, or irregular damage. To address these issues, image processing strategies were implemented to inpaint the damaged mask images. The process began with the binary conversion of image pixels, where a threshold of 128 was used to convert grayscale values from the range of 0 to 255 to the binary values of 0 and 1. Subsequently, hole-filling and despeckle techniques were applied to enhance the quality of the mask images. The visualization of images before and after hole-filling and despeckle processes are shown in Figure 4A–D.

Once the mask image was repaired, TKV could be determined. We began by separating the left and right kidneys and proceeded to calculate the pixel count of the kidney area in each slice. Relevant attributes from the DICOM tags were collected for TKV calculation. The PixelSpacing provided the conversion ratio between pixels and the actual area, while the slice thickness represented the actual thickness of each slice.

Subsequently, the respective volumes of the left and right kidneys were calculated based on these attributes. Lastly, the TKV was computed by summing the volumes of the right and left kidneys together.

### 2.9. Experiment Environment

The hardware configuration utilized for this study consisted of an Intel i7-9700KF CPU paired with 16GB of DDR4 RAM, complemented by the Nvidia RTX2080 8G graphic card. Our study utilized Python as the programming language and TensorFlow with Keras as the deep learning framework.

### 2.10. Statistics

For data exhibiting a normal distribution, *Student’s t-test* was applied for single variable assays. Alternatively, data not conforming to a normal distribution were analyzed using the Mann–Whitney test for single variable assays. A *p*-value of less than 0.05 is considered statistically significant.

## 3. Results

### 3.1. Images Collection

**Figure 5:**
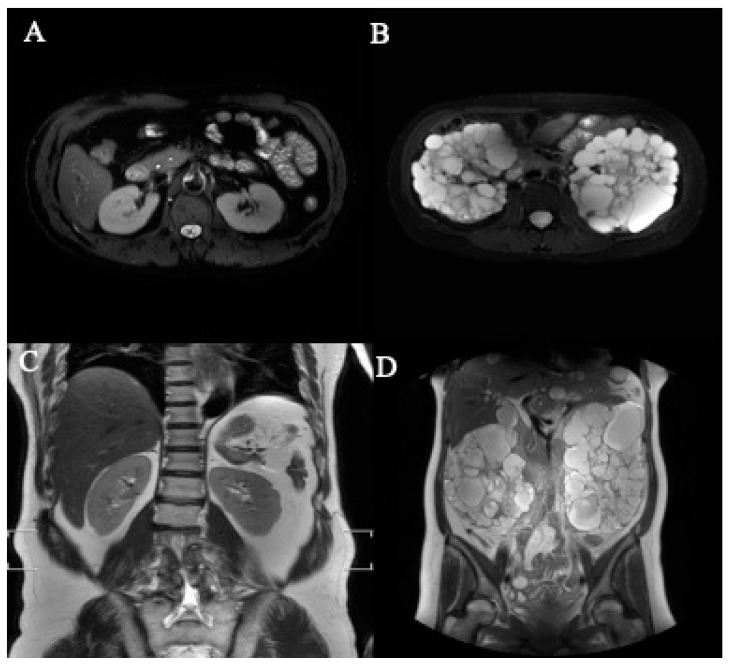
The representative axial- and coronal-section MRI images of kidneys. (**A**) Normal kidney (axial section), (**B**) polycystic kidney (axial section), (**C**) normal kidney (coronal section), (**D**) polycystic kidney (coronal section).

A total of 40 MRI image sets were collected for the study, comprising 30 sets from patients with ADPKD and 10 sets from healthy participants (Table 1). The axial and coronal sections, both with and without ADPKD, are summarized in Table 1. Representative images depicting axial and coronal sections, both with and without ADPKD, are illustrated in Figure 5.

For the training dataset, we randomly allocated 21 ADPKD participants and nine healthy participants. The remaining 10 participants, comprising nine with ADPKD and one healthy participant, were assigned to the testing dataset. Table 2 offers an overview of the patient-wise data segmentation. Comprehensive details regarding the training and testing images are presented in Table 3.

**Figure 6:**
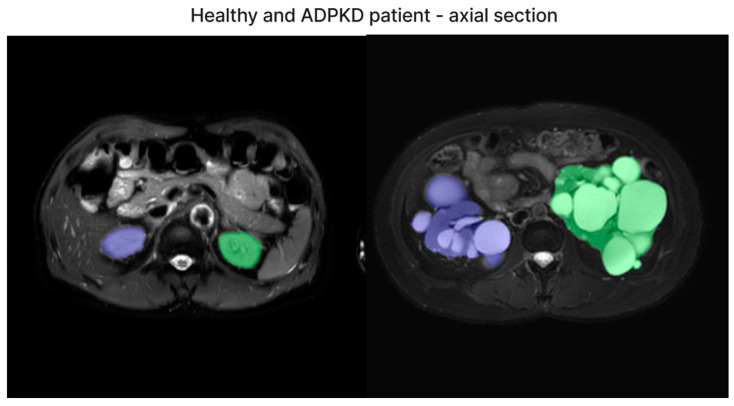
The representative image of axial section visual segmentation.

**Figure 7:**
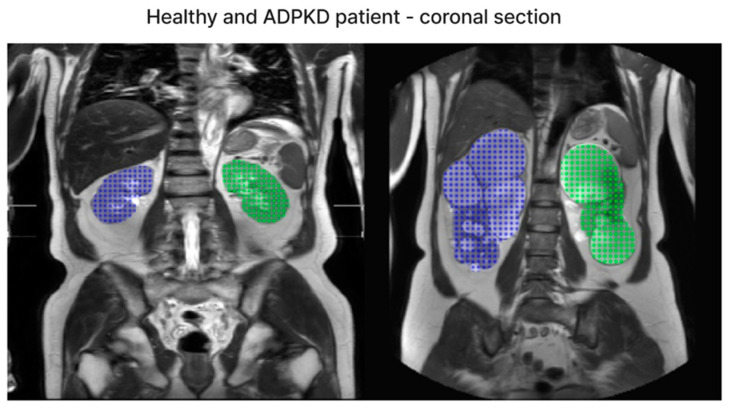
The representative image of coronal section visual segmentation.

We have included both healthy and ADPKD results for coronal and axial views in Figure 6 and Figure 7.

### 3.2. Validation and Results Comparison

In the testing dataset, there were a total of 10 participants, with nine diagnosed with ADPKD and one classified as a healthy participant. This dataset comprised both axial-section and coronal-section images. The training process involved training on both axial and coronal sections using the U-net model for 500 epochs. The training of the axial-section model reached an early stopping point at the 105th epoch, with validation results indicating a DSC of 0.89, whereas the training of the coronal-section model concluded at the 103rd epoch, achieving a best validation DSC of 0.79. The performance results of the axial-section and coronal-section models are summarized in Table 4 and Table 5, respectively.

Although all the images were derived from the same cohort, there were slight variations in the ground TKV measurements between coronal- and axial-section images. The mean TKV measured from the testing dataset by medical professionals (ground truth) was 1501.8 ± 965.8 mL using axial-section images and 1740.3 ± 1172.2 mL using coronal-section images, respectively (*p* = 0.73) (Table 4 and Table 5). In comparison, the mean TKV estimated by the deep learning model was 1536.3 ± 958.7 mL for axial-section images and 1636.2 ± 964.7 mL for coronal-section images (*p* = 0.85) (Table 4 and Table 5). The mean difference in TKV between medical professionals and the deep learning model was 44.2 ± 58.7 mL (3.95 ± 4.14%) for axial-section images (*p* = 0.8) and 329.1 ± 352.6 mL (21.6 ± 22.4%) for coronal-section images (*p* = 0.9), respectively. For reference, we also implemented the mid-slice method and found that it resulted in a much greater difference. The mean difference in TKV between medical professionals and the mid-slice method was 945.8 ± 747.9 mL (55.8 ± 24.9%) for axial-section images and 1095.4 ± 731.3 mL (59.8 ± 14.6%) for coronal-section images. Collectively, regardless of whether axial- or coronal-section images were used, there was no statistical difference in TKV measurement between medical professionals and our deep learning model. However, a larger difference was observed between medical professionals and the deep learning model when coronal-section images were used.

### 3.3. Accuracy of Segmentation

By employing the DSC and Jaccard coefficient metrics, we quantified the accuracy of the image segmentation process for both the coronal-section and the axial-section models. The Jaccard coefficient was calculated using the formula:

Jaccard = TP/(TP + FP + FN)

**Figure 8:**
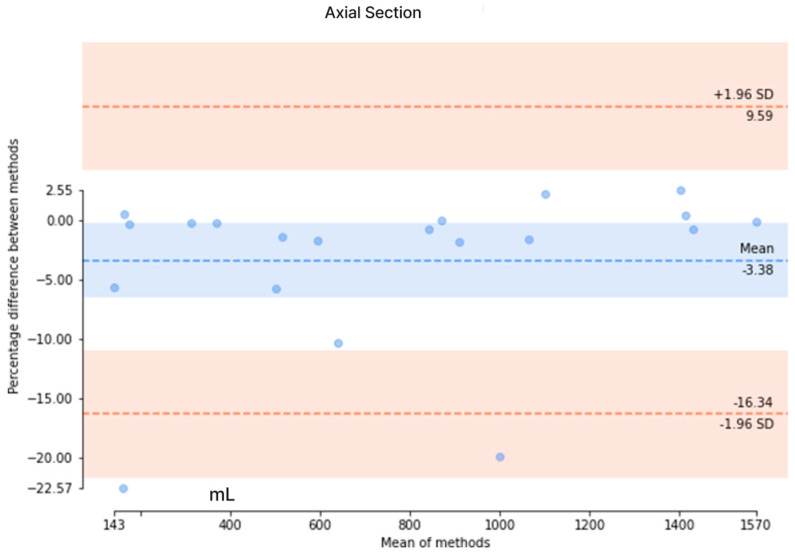

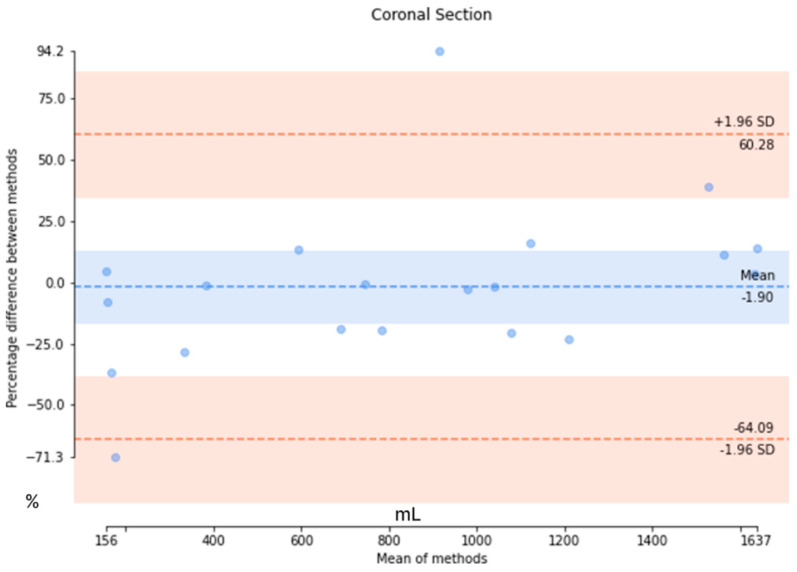
The Bland–Altman analysis to evaluate the agreement between the ground truth values and the predicted values in axial and coronal sections. (**Upper**) The Bland–Altman analysis of the axial section and (**Lower**) the Bland–Altman analysis of the coronal section.

The results from the axial-section model revealed a mean DSC of 0.89 ± 0.27 and an average patient-wise Jaccard coefficient of 0.86 ± 0.27. The mean difference of the axial-section model was determined to be 44.2 ± 58.7 mL, with a mean percentage difference of 3.95 ± 4.14%. Conversely, the mean DSC of the coronal-section model was 0.82 ± 0.29, with a mean Jaccard coefficient of 0.77 ± 0.31. The mean error for the coronal-section model was 329.1 ± 352.6 mL, and the mean percentage error was 21.6 ± 22.4%. These metrics indicated that the axial-section model outperformed the coronal-section model across all evaluation measures. Additionally, Bland–Altman analysis was conducted to evaluate the agreement between the ground truth values of TKV and the predicted values of the axial- and coronal-section models, as depicted in Figure 8. For the axial-section model comparison, a bias of −3.38% was observed, with upper and lower limits of agreement at 9.59 and −16.38, respectively. Similarly, the coronal-section model comparison resulted in a bias of −1.90%, with upper and lower limits of agreement at 60.28 and −64.09, respectively. While the coronal-section model demonstrates a minimal average difference (−1.90%) between predicted results and ground truth values, the substantial variability in the limits of agreement highlights the model’s inconsistent precision, with individual predictions often straying significantly from the actual values. This inconsistency underscores the need for caution when interpreting the model’s results.

Additionally, we have compared our method with other deep learning-based TKV methods. In our study, we have explored both the coronal- and axial-section images to calculate the TKV. From the comparison in Table 6, it is evident that 2D U-Net is commonly utilized for TKV calculation across various modalities, including 3D ultrasound, CT, and MRI. While a direct comparison of Dice scores is challenging due to the use of different datasets, our model outperformed both 3D ultrasound-based [32] and CT-based [28] models in terms of Dice score. When compared to other methods, our approach produced results comparable to other 2D U-Net models. In our coronal-section model, the limits of agreement ranged from −64.09% to +60.28%, while in the axial-section model, they ranged from −16.38% to +9.59%. In comparison, Sharma’s study [28] reported limits of agreement of −18.6% to +20.3% for study 1 and −29.6% to +38.9% for studies 2 and 3. Our axial-section model demonstrates narrower limits of agreement than both study 1 and studies 2 and 3, indicating better agreement and reduced variability in predictions relative to the ground truth. However, the coronal-section model exhibited much wider limits of agreement, suggesting greater variability and less consistent alignment between its predictions and the actual values.

## 4. Discussion

ADPKD stands as the most prevalent inherited renal disease, culminating in cyst formation and eventual ESRD. TKV serves as a crucial indicator of ADPKD prognosis and treatment response, underscoring the importance of accurate TKV estimation. Traditionally, TKV measurement relied on manual assessments by medical professionals, which proved to be laborious and time-intensive. However, AI, particularly deep learning, offers a promising solution.

Different imaging modalities can be utilized for TKV measurement, with T2-weighted MRI emerging as a superior option due to its accuracy, minimal radiation exposure, and avoidance of potential contrast-associated nephrotoxicity [9]. Employing image segmentation techniques such as V-Net and U-Net on MRI images has shown significant promise in enhancing TKV measurement accuracy [27,38]. This application efficiently enhances TKV measurement for treatment and outcome evaluations. In our study, we introduced the U-net framework to provide precise TKV calculations from MRI data [21]. We trained the model using MRI images from 30 ADPKD patients and 10 healthy participants. Our methodology aligns with the broader trend of leveraging deep learning for medical image analysis [39]. Moreover, our specific approach of using the U-net framework for TKV measurement offers a novel perspective.

In our pilot study, we utilized both axial- and coronal-section images for training. We observed no significant difference between medical professionals and AI-assisted TKV measurements, suggesting that either our AI model’s performance was comparable to that of medical professionals or the difference was undetectable with our sample size. Although no significant difference in TKV results was found between axial- and coronal-section models, the axial-section model exhibited better performance in both variances and accuracy. However, this preference for axial-section images might introduce a bias in our method or indicate that our model is specifically suitable for axial-section images. The differences between axial- and coronal-section images might suggest that TKV measurement discrepancies could naturally arise based on image orientation. This would also suggest that the presence of measurement bias between medical professionals and AI models based on either axial or coronal sections was inevitable. Indeed, consistency in the application of the measurement method across all participants/images is crucial for ensuring the validity and reliability of the prediction model and its results. Consistency helps to mitigate potential biases and ensure that the model’s performance is accurately assessed based on standardized criteria. Therefore, maintaining consistency in the measurement process is paramount in producing valuable and reliable predictions that can be effectively utilized in clinical practice.

Our results align with the growing body of evidence supporting the use of AI in healthcare, particularly in medical imaging [17,18,19,20]. The DSC and Jaccard coefficient metrics indicate that our model’s performance is comparable to traditional methods of TKV calculation [40]. Previous studies have utilized various methods to calculate kidney volumes in ADPKD patients, with notable achievements. For instance, the Spatial Prior Probability Map (SPPM) model achieved an average DSC of 0.86 [28]. In our study, we achieved a slightly higher DSC of 0.89 ± 0.27 and an average patient-wise Jaccard coefficient of 0.86 ± 0.27. These results are particularly relevant given the significance of TKV as a prognostic indicator in ADPKD, and accurate measurement should be the basis of all methods. Additionally, our study contributes to the literature by comparing the performance of axial- and coronal-section models, a comparison less frequently made in existing research. Moreover, our Bland–Altman analysis demonstrated good agreement between ground truth values and our model’s predictions, indicating the reliability of our method in clinical settings. The automation of TKV measurement through deep learning has the potential to revolutionize ADPKD management, offering a valuable tool for monitoring disease progression and evaluating treatment efficacy. Although we concluded that axial-section MRI images provide greater accuracy compared to coronal-section images, studies utilizing axial-section MRI (including ours and those by Goel et al.) [35] reported an average DSC of 0.92, which is comparable to the average DSC of 0.91 from studies using coronal-section MRI images [33,36,37]. Regardless of whether axial or coronal sections are used, MRI remains the preferred imaging modality recommended by the Consortium for Radiologic Imaging Studies of Polycystic Kidney Disease (CRISP) [41].

While the study is promising, there are several limitations that should be acknowledged. The relatively small dataset of 40 participants may limit the generalizability of our findings. Additionally, the lack of a head-to-head comparison with other TKV measurement methods hinders the assessment of our model’s relative advantages or efficiency. Relying solely on a single type of deep learning model (U-net) may also restrict our study’s performance despite the promising results reported in previous studies [30]. Furthermore, our focus on MRI data overlooks other imaging techniques, such as CT, which are also used for kidney volume estimation. Future research should address these limitations by including larger and more diverse patient populations and conducting comparative studies of TKV measurement using different methods. Additionally, further exploration of the clinical implications, integration into current clinical workflow, and evaluation of cost-effectiveness are warranted. Clinical trials assessing the real-world applicability of our model could provide valuable insights into its utility and potential impact on patient care.

## 5. Conclusions

Our study demonstrates the feasibility and effectiveness of using a deep learning model for TKV measurement in patients with ADPKD using MRI data. The results indicate that the deep learning model performs comparably to medical professionals, regardless of whether axial- or coronal-section images are used. However, the model showed superior accuracy and consistency with axial-section images, suggesting that image orientation affects measurement reliability. These findings underscore the potential of deep learning in enhancing TKV measurement and improving patient management in ADPKD. Further research with larger datasets and comparative studies is needed to optimize and validate this approach for broader clinical applications.

## Figures and Tables

**Table 1:** The training and testing datasets of TKV measurement.

Dataset	ADPKD (n)	Non-ADPKD (n)	Total (n)
Training set	21	9	30
Testing set	9	1	10
Total (n)	30	10	

**Table 2:** The information of MRI images.

	Participant (n)	Axial-Section Images (n)	Coronal-Section Images (n)
ADPKD	30	1572	1265
Non-ADPKD	10	437	271

**Table 3:** The details of MRI images in ADPKD and non-ADPKD participants.

Dataset	Total Images Axial/Coronal (n)	ADPKD Images Axial/Coronal (n)	Non-ADPKD Images Axial/Coronal (n)
Training set	1483/1127	1094/883	389/244
Testing set	526/409	478/382	48/27
Total images axial/coronal (n)	2009/1536	1572/1265	437/271

**Table 4:** The performance of TKV testing cases (axial-section model).

Participants	Ground Truth (mL)	Our Method (mL)	Diff. (mL)	Diff. (%)
Participant 1	2992.23	3004.72	12.49	0.42
Participant 2	1078.51	1117.47	38.96	3.61
Participant 3	1121.51	1195.16	73.65	6.57
Participant 4	2169.31	2162.39	6.92	0.32
Participant 5	1740.32	1763.23	22.91	1.32
Participant 6 (Non-ADPKD)	316.14	324.86	8.72	2.76
Participant 7	310.54	346.36	35.82	11.53
Participant 8	2836.07	2794.3	41.77	1.47
Participant 9	1770.83	1970.3	199.47	11.26
Participant 10	682.89	684.51	1.62	0.24
Mean ± SD	1501.8 ± 965.8	1536.3 ± 958.7	44.2 ± 58.7	3.95 ± 4.14

**Table 5:** The performance of TKV testing cases (coronal-section model).

Participants	Ground Truth (mL)	Our Method (mL)	Diff. (mL)	Diff. (%)
Participant 1	3573.87	2753.84	820.03	22.95
Participant 2	1328.97	1614.76	285.79	21.5
Participant 3	1374.44	1303.08	71.36	5.19
Participant 4	2557.89	1515.7	1042.19	40.74
Participant 5	1995.45	2039.09	43.64	2.19
Participant 6 (Non-ADPKD)	248.8	434.7	185.9	74.72
Participant 7	312.48	317.74	5.26	1.68
Participant 8	3310.56	3078.19	232.37	7.02
Participant 9	2034.05	2540.41	506.36	24.89
Participant 10	666.63	764.95	98.32	14.75
Mean ± SD	1740.3 ± 1172.2	1636.2 ± 964.7	329.1 ± 352.6	21.6 ± 22.4

**Table 6:** The comparison with other deep learning models.

Study	Modality	Method	No. of Patients	Dice Score
Jagtap [32]	3D-Ultra sound	2D U-Net	22	0.80
Sharma [28]	CT	2D VGG-16 FCN	125	0.86
Raj [33]	MRI-Coronal	2D Attention U-Net	100	0.922
Taylor [34]	MRI	3D U-Net	227	0.96
Goel [35]	MRI Axial T2	2D U-Net + EfficientNet encoder	173	Test set 0.95
Kline [36]	MRI Coronal T2 +/− fatsat	2D U-Net + ResNet-like encoder	60	1st Reader: 0.86 2nd Reader: 0.84
Van Gastel [37]	MRI Coronal T2 fatsat	2D U-Net	145	0.96
Our Method	MRI Axial + Coronal T2	2D U-Net	40	0.89/0.82

## Data Availability

Data are included in the article and are available upon request.
